# DeepVAQ : an adaptive deep learning for prediction of vascular access quality in hemodialysis patients

**DOI:** 10.1186/s12911-024-02441-2

**Published:** 2024-02-12

**Authors:** Sarayut Julkaew, Thakerng Wongsirichot, Kasikrit Damkliang, Pornpen Sangthawan

**Affiliations:** 1 College of Digital Science, Prince of Songkla University, Hat Yai, Songkhla, Thailand; 2 Division of Computational Science, Faculty of Science, Prince of Songkla University, Hat Yai, Songkhla, Thailand; 3 Department of Medicine, Division of Nephrology, Faculty of Medicine, Prince of Songkla University, Hat Yai, Songkhla, Thailand

**Keywords:** Deep learning, Hemodialysis, Vascular access

## Abstract

**Background:**

Chronic kidney disease is a prevalent global health issue, particularly in advanced stages requiring dialysis. Vascular access (VA) quality is crucial for the well-being of hemodialysis (HD) patients, ensuring optimal blood transfer through a dialyzer machine. The ultrasound dilution technique (UDT) is used as the gold standard for assessing VA quality; however, its limited availability due to high costs impedes its widespread adoption. We aimed to develop a novel deep learning model specifically designed to predict VA quality from Photoplethysmography (PPG) sensors.

**Methods:**

Clinical data were retrospectively gathered from 398 HD patients, spanning from February 2021 to February 2022. The DeepVAQ model leverages a convolutional neural network (CNN) to process PPG sensor data, pinpointing specific frequencies and patterns that are indicative of VA quality. Meticulous training and fine-tuning were applied to ensure the model’s accuracy and reliability. Validation of the DeepVAQ model was carried out against established diagnostic standards using key performance metrics, including accuracy, specificity, precision, F-score, and area under the receiver operating characteristic curve (AUC).

**Result:**

DeepVAQ demonstrated superior performance, achieving an accuracy of 0.9213 and a specificity of 0.9614. Its precision and F-score stood at 0.8762 and 0.8364, respectively, with an AUC of 0.8605. In contrast, traditional models like Decision Tree, Naive Bayes, and kNN demonstrated significantly lower performance across these metrics. This comparison underscores DeepVAQ's enhanced capability in accurately predicting VA quality compared to existing methodologies.

**Conclusion:**

Exemplifying the potential of artificial intelligence in healthcare, particularly in the realm of deep learning, DeepVAQ represents a significant advancement in non-invasive diagnostics. Its precise multi-class classification ability for VA quality in hemodialysis patients holds substantial promise for improving patient outcomes, potentially leading to a reduction in mortality rates.

## Background

The number of end-stage kidney disease (ESKD) patients on hemodialysis (HD) has been rapidly increasing worldwide and is accompanied by the high burden of HD vascular access (VA)-related complications. Globally, the HD patients comprise approximately 69% of all individuals receiving renal replacement therapy and 89% of those undergoing dialysis procedures [1]. In Southeast Asia, the prevalence rate (incidence per million persons) of HD patients is approximately 473.3 compared with higher and lower income countries at 305.8 and 167.5, respectively. The prevalence rates are expected to increase by 10% over five years [2, 3]. VA serves as a critical lifeline for HD patients, providing the necessary means for their dialysis treatment [4]. There are two commonly used types of VAs: AVF (Arteriovenous Fistula) and AVG (Arteriovenous Graft). AVF involves directly connecting an artery and a vein, while AVG utilizes a synthetic graft to establish the connection. Maintaining a properly functioning VA is of utmost importance for effective HD management. Any malfunction in the VA can lead to dialysis insufficiency and significantly increase the morbidity and mortality risks for HD patients. Stenosis and thrombosis are the primary causes of VA dysfunction, necessitating timely detection and intervention to ensure the well-being and survival of HD patients [5].

The prevalent method for detecting VA stenosis and thrombosis in HD patients involves measuring VA blood flow via ultrasound dilution (VABF-UD), a technique considered the gold standard in HD treatment. This method has notably improved patient care by facilitating timely interventions, maintaining VA functionality, and mitigating risks associated with inadequate dialysis [6, 7]. However, the broader application of VABF-UD is constrained by factors such as cost and limited accessibility, particularly in developing or under-developed countries [8]. These barriers restrict its integration into routine clinical practice, limiting its benefits to a broader patient population.

In this study, we propose the utilization of low-cost PPG sensors, known for their effectiveness in capturing biosignals, combined with a deep learning model based on fine-tuned parameters in a convolutional neural network (CNN) architecture called “DeepVAQ”. The resulting DeepVAQ model demonstrates remarkable accuracy in early detection and prediction of VA quality in HD patients, offering a promising and effective approach for forecasting VA quality. This study contributes to the development of screening tests specifically tailored to evaluate VA quality in HD patients, enhancing early detection, patient care, and treatment outcomes.

## Literature review

Recent advancements in monitoring health conditions and diagnosing diseases have increasingly capitalized on the integration of sensor technology with machine learning. Prominent among these technologies are Photoplethysmography (PPG) sensors, non-invasive devices that monitor blood volume variations in the vascular system [9]. These sensors function by detecting changes in light absorption or reflection, which facilitates the estimation of essential physiological parameters such as pulse rate, blood flow, and oxygen saturation. These parameters are crucial for non-invasive health monitoring [10, 11]. The application of wearable PPG sensors in healthcare has been extensively documented [12], particularly their utility in monitoring blood circulation changes, exemplified by their use in continuous heart rate monitoring. These sensors are also promising for the early detection of cardiovascular diseases and for real-time monitoring in clinical environments. The versatility of PPG sensors across various healthcare applications, including their use in vascular occlusion training, is well-established [13, 14]. Advances in PPG signal analysis for biomedical purposes have seen considerable progress, especially with the incorporation of sophisticated analytical methods and various sensor types. Deep learning algorithms, particularly the CNN-LSTM model applied to PPG signals, have shown to outperform other algorithms [15–17]. This highlights the potential for further research into model architectures, hyperparameters, and time–frequency representations to refine PPG signal analysis. It is imperative to note the unique nature of vascular access in HD patients, which demands specialized consideration compared to other patient groups. While PPG sensors and deep learning techniques hold significant promise in healthcare, targeted research on their efficacy in evaluating VA quality for HD patients is scant. Advancing this line of inquiry is vital to harness the full capabilities of these technologies in enhancing HD patient care.

## Methods

**Fig. 1:**
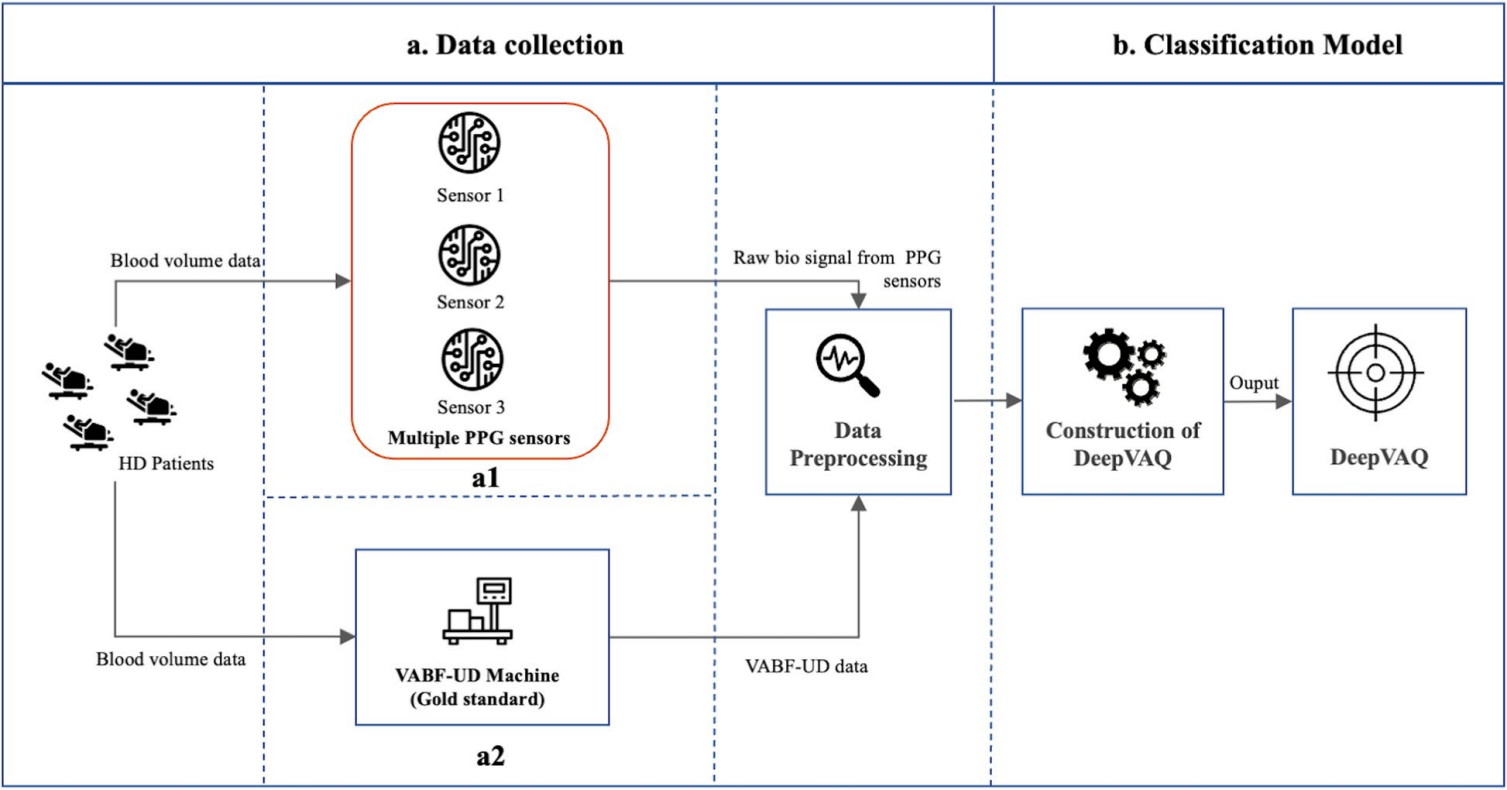
Research experimental design

The experiment comprised two main sections: data collection (a) and classification (b) as shown in Fig. 1.

Data Collection (a): Data were gathered from 398 HD patients with both types of VA, Arteriovenous Fistula (AVF) and Arteriovenous Graft (AVG). The patient cohort included 246 males (61.81%) and 152 females (38.19%), with ages ranging from 29 to 90 years and an average age of 63 years. Among these patients, 155 (38.94%) had an AVG and 243 (61.06%) had an AVF. The VA locations were on the left arm for 276 patients (75.20%) and on the right arm for 91 patients (24.80%), with 141 (36.34%) on the upper arm and 247 (63.66%) on the forearm. This demographic and clinical information is detailed in Table 1. The data collection involved two sources: multiple PPG sensors (a1) and the VABF-UD machine (a2). For the PPG data collection, we employed the SEN0203 model PPG sensors [18], equipped with the SON1303 IC/Module. Three PPG sensors were strategically placed over each patient's VA site on the skin by expert staff specializing in VA, to ensure accurate positioning and data collection. This placement was standardized to ensure consistent data quality, with sensors attached using pads, and the skin area was cleaned and prepared. The PPG sensors recorded signals for a continuous period of 5 min before the patients' dialysis sessions. Concurrently, VABF data (ml/min) were collected from the patients using a device manufactured by Transonic Systems Inc [19] during their dialysis procedures.

**Table 1:** Demographic and clinical characteristics of HD patients

Variable	Category	HD patients (*n)*	HD patients (*%*)
Gender	Male	246	61.81%
	Female	152	38.19%
Age	Maximum	90	
	Minimum	29	
	Average	63	
Type of Vascular Access	AVG	155	38.94%
	AVF	243	61.06%
Arm	Left	276	75.20%
	Right	91	24.80%
Location on arm	Upper arm	141	36.34%
	Forearm	247	63.66%

Classification Model (b): In this section, DeepVAQ was constructed by conducting a series of experiments with different parameter settings. Each experiment underwent comprehensive evaluation using multiple performance measurements to identify the optimal parameters for the proposed model.

In previous studies [20, 21], researchers have established optimal criteria for detecting stenosis and thrombosis. Among the various measurement techniques, the VABF (vascular access blood flow) standard has been identified as the most effective predictor of impending stenosis and thrombosis. We utilized a combination of the aforementioned VABF criteria to classify the dataset and predict the quality of VA. Through the use of statistical methods [22], we determined the range of VABF rates associated with different VA quality classes, which are summarized in Table 2.

**Table 2:** Classification of VA Quality based on VABF Rate

Class	VA quality	VABF rate Minimum (ml/min)	VABF rate Maximum (ml/min)	Description
1	Poor	0	450	High chance of VA stenosis and thrombosis
2	Below Medium	451	900	Prone to VA stenosis and thrombosis
3	Medium	901	1,350	Good VA quality
4	Good	1,351	1,800	High VA Quality
5	Excellent	$\ge$ 1,801		Highest VA Quality

The classification allows for the identification of VA conditions associated with a high chance of stenosis and thrombosis (Class 1), as well as those prone to such complications (Class 2). Additionally, it distinguishes between VA with good (Class 3), high (Class 4), and excellent (Class 5) quality.

### Datasets and preprocessing

**Fig. 2:**
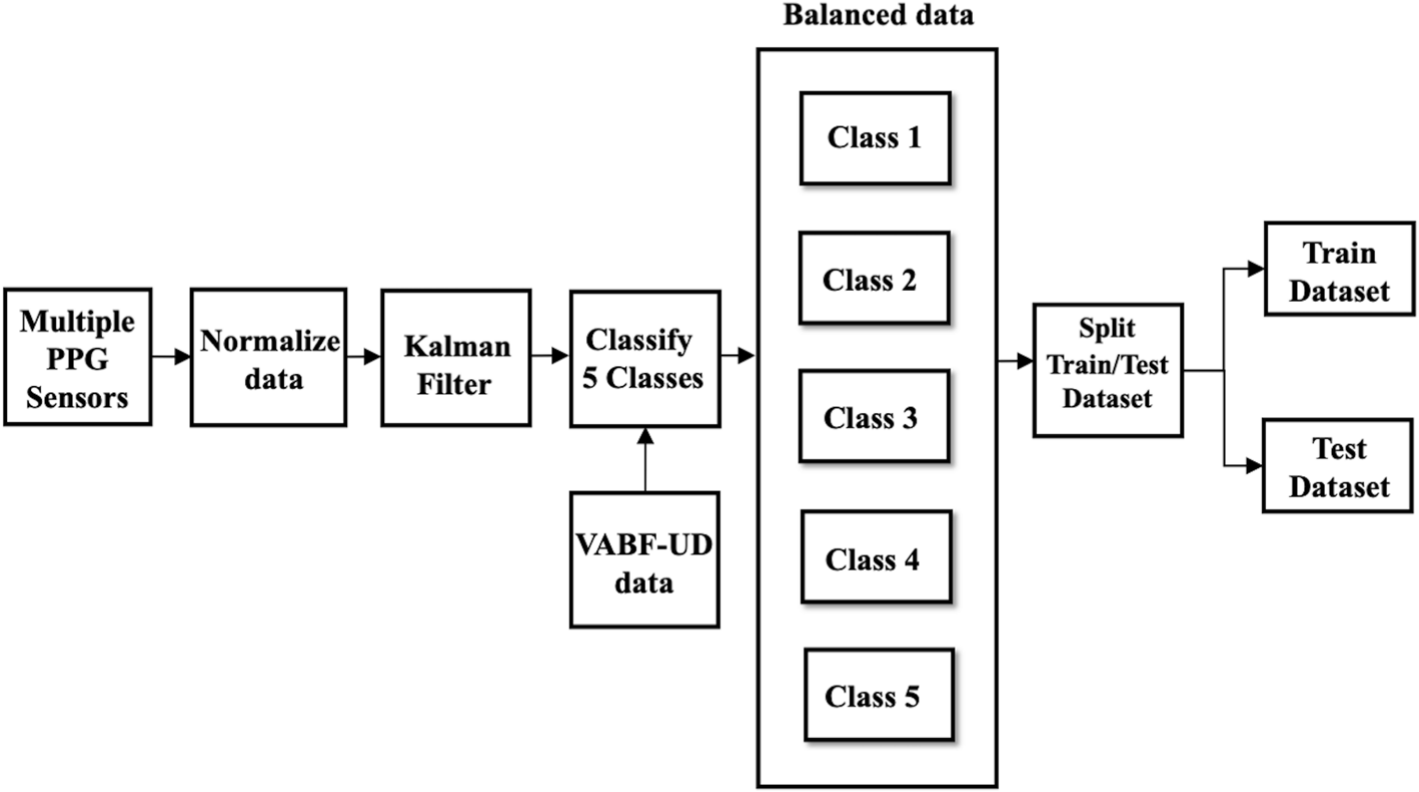
Dataset and preprocessing

According to Fig. 2, PPG sensors were utilized to gather data from HD patients, resulting in a total of 27,000 measurements (9,000 values per sensor) in a 1D-formatted data representation. This corresponds to an approximate sampling rate of 30 values per second. To ensure consistent scaling of the features, the raw dataset was normalized using the min–max method, which rescales the values between 0 and 1 [23]. The normalization process is governed by Eq. (1):

1 $${{\text{x}}}^{\mathrm{^{\prime}}}= \frac{{{\text{x}}}_{{\text{i}}}-{{\text{x}}}_{{\text{mn}}}}{{{\text{x}}}_{{\text{m}}}-{{\text{x}}}_{{\text{mn}}}}$$

where ${{\text{X}}}_{{\text{mn}}}$ denotes minimum value, ${{\text{X}}}_{{\text{m}}}$ denotes maximum value, ${{\text{X}}}_{{\text{i}}}$ denotes input value, and ${{\text{X}}}^{\mathrm{^{\prime}}}$ denotes normalized data.

To mitigate noise from the PPG sensors, we employed a Kalman filter, which utilizes a linear estimator to estimate the value of an unknown variable over time by considering previous data and weighing it against the actual data [24]. Applying the Kalman filter significantly enhances the accuracy and reliability of the PPG sensor data.

To facilitate the classification process, the PPG sensor data was assigned labels based on five distinct classes corresponding to the range of VA quality, as presented in Table 2. These classes serve as reference points for categorizing the VA quality based on the specific range of values associated with the PPG sensor measurements.

The collected dataset initially had an imbalance, which could introduce bias and impact prediction accuracy. To address this issue and avoid overfitting, we employed the Synthetic Minority Oversampling Technique (SMOTE) [25]. SMOTE, a widely used method, creates new synthetic samples for the minority class by utilizing a k-nearest neighbor algorithm. By augmenting the representation of the minority class with these synthetic samples, we aimed to rebalance the dataset and improve the model's predictive capabilities for both majority and minority classes. This adjustment through SMOTE ensured a more balanced representation of each class in the dataset, reducing bias and enhancing overall performance. As a result, all classes consisted of 125 samples, resulting in a total of 625 samples in the dataset. Table 3 presents the number of samples for each class before and after the application of SMOTE.

**Table 3:** Classes distribution before and after SMOTE sampling

Class	VA quality	Number of samples	
		**Before SMOTE**	**After SMOTE**
1	Poor	22	125
2	Below Medium	125	125
3	Medium	108	125
4	Good	55	125
5	Excellent	28	125

We further partitioned the dataset into training and testing sets using a 70:30 ratio. The training set, comprising 70% of the data, was used for model training and parameter optimization. The remaining 30% of the data was reserved for evaluating the trained model's performance on unseen samples.

Table 4 illustrates the distinct waveforms of VA quality data captured by the PPG sensor. The waveforms exhibit intricate and diverse patterns within a frequency range of 0–300. These patterns vary significantly across different classes, highlighting the complexity and uniqueness of each VA quality category.

**Table 4:** Waveform of VA quality data from PPG sensor

Class	VA quality	Waveform
1	Poor	
2	Below Medium	
3	Medium	
4	Good	
5	Excellent	

### DeepVAQ model construction

**Fig. 3:**
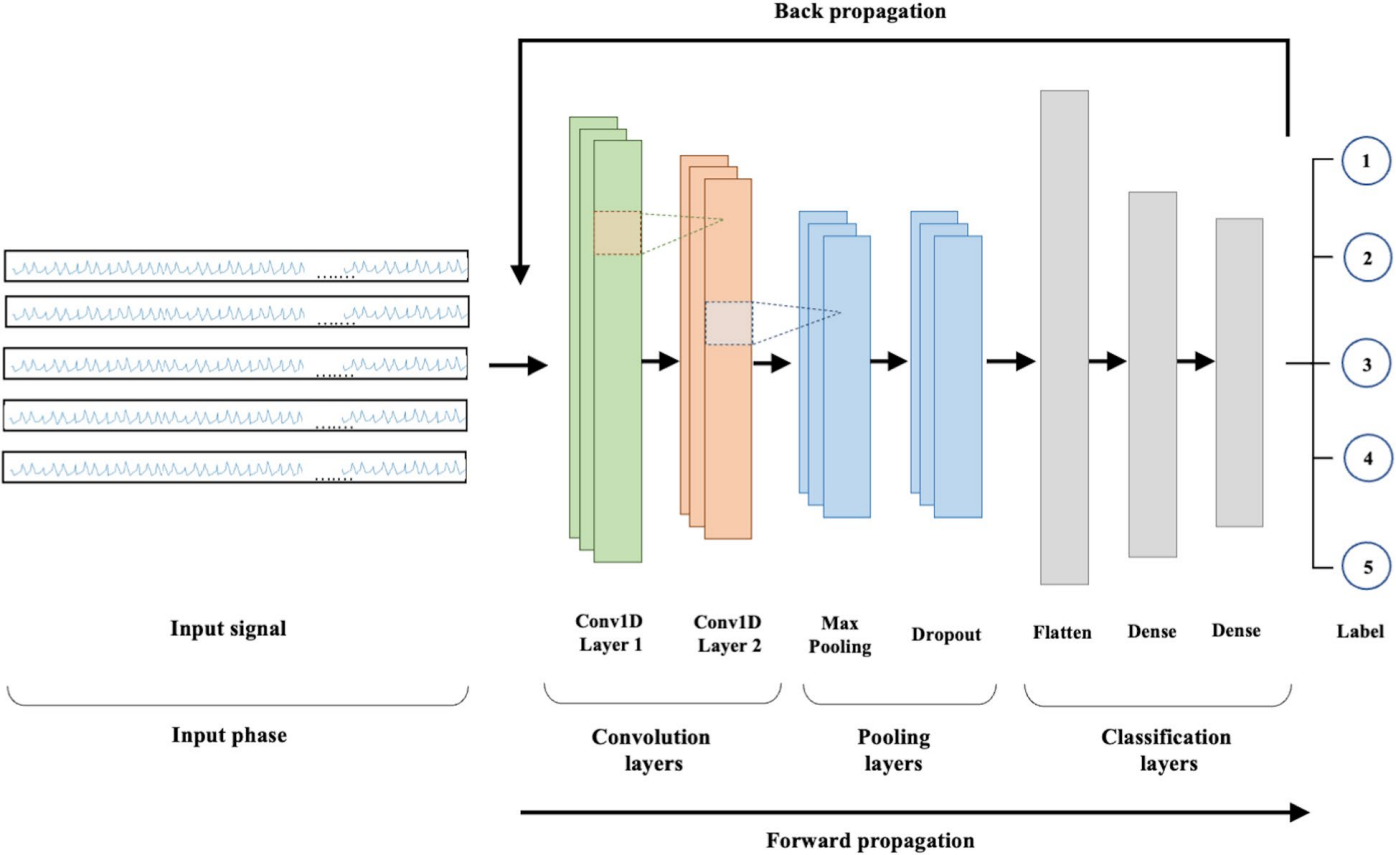
Convolution neural network architecture

The construction of the DeepVAQ model employed in this study can be described as a highly sophisticated and intricate model as show in Fig. 3, surpassing the simplicity of CNN architectures. It stands out due to its extensive complexity and advanced design elements. One notable aspect is the utilization of two layers of 1D-CNN, which significantly enhances its capacity to extract meaningful features which are then converted into vectors based on Eq. (2). By incorporating multiple filters such as {8, 16, 32} and varying kernel sizes {3, 5, 7} [26, 27], the model becomes capable of capturing a wide range of intricate patterns and nuanced details present in the data.

2 $$y\left(n\right)= \left\{\begin{array}{cc}{\sum }_{i=0}^{k}\,x\left(n+i\right)h\left(i\right) & if n=0.\\ {\sum }_{i=0}^{k}x\left(n+i+\left(s-1\right)\right)h\left(i\right),& otherwise.\end{array}\right\}$$

Where x is the input to the convolution layer of length n, h the kernel of length k, and s represents the kernel window shift positions (number of strides) after each convolution.

The DeepVAQ model's sophistication is enhanced through customized parameter tuning. The selection of appropriate filters and kernel sizes optimizes feature extraction, which is crucial for discerning and classifying diverse data patterns. The incorporation of the Leaky ReLU activation function [28] introduces a small negative slope for negative inputs, thereby improving the model's ability to handle complex data patterns. The model is further optimized using the Adam optimizer [29]. This algorithm adapts the learning rate based on the gradient of the loss function, promoting faster convergence and improved performance. The dynamic adjustment of the learning rate by the Adam optimizer aids in efficiently navigating the loss landscape, optimizing parameter values for better training outcomes.

Within its convolutional architecture, DeepVAQ incorporates max pooling and dropout layers. Max pooling, with a 2 × 2 size, reduces the dimensionality of the feature maps, focusing on important features. The inclusion of a dropout layer, set at a rate of 0.5, acts as a regularization technique. It prevents overfitting by randomly dropping neuron outputs during training, thereby enhancing the model's generalization and adaptability to varied datasets.

The classification layers of the model are instrumental for multi-class classification. These layers consolidate feature vectors from preceding layers into a synthesized form for processing in a dense layer, which is pivotal for final classification. The use of the one-hot encoding technique [30] ensures accurate differentiation among the five VA quality classes.

In the training process, the DeepVAQ model utilizes the cross-entropy loss function [31], a widely-accepted measure for multi-class classification tasks. This loss function quantifies the dissimilarity between the predicted class probabilities and the actual ground truth labels, serving as a crucial metric for evaluating the model's performance and guiding the optimization process. The number of training epochs is strategically set to balance the model's complexity and efficiency.

### DeepVAQ model evaluation

The DeepVAQ model utilized a ten-fold cross-validation approach on the test set to validate its performance across multiple iterations, enhancing the reliability of the results. The evaluation metrics included accuracy, sensitivity, specificity, precision, and F-score, which are commonly used classification performance measurements [32]. These metrics provide a comprehensive assessment of the model's predictive capabilities and its ability to correctly classify different VA quality classes. The evaluation was conducted on the test set, and the mean and standard deviation of the performance measurements are presented in Table 5.

**Table 5:** Performance measurements

Performance measurements	Formula
Accuracy	(TP + TN)/(TP + FP + TN + FN)
Sensitivity	TP/(TP + FN)
Specificity	TN/(TN + FP)
Precision	TP/(TP + FP)
F-Measure	$(2 \times \mathrm{Precision }\times \mathrm{ Recall})/({\text{Precision}}+{\text{Recall}})$

*TP* True Positive, *TN* True Negative, *FP* False Positive, *FN* False Negative

The development of the DeepVAQ model was built using the Keras high-level API [33] in Python, providing a powerful and user-friendly framework for deep learning. The experiments were conducted on a robust computational infrastructure, featuring an Intel Core i7-7700 processor with a clock speed of 3.60 GHz, 16-GB DDR4 RAM, and a 512-GB solid-state drive (SSD). Complementing the hardware, an NVIDIA Quadro-620 GPU with 2-GB GDDR5 memory was employed for efficient processing of the complex computations involved in model training and evaluation.

## Results

The DeepVAQ model was evaluated through a series of experiments, where different parameter settings, filter sizes, and kernel sizes were tested. In each experiment, the filter sizes (i) were set to 8, 16, and 32, while the kernel sizes (j) were set to 3, 5, and 7. The results of these experiments are presented in Table 6. Among all the experiments, exp((16,5)) achieved the highest average performance across all measurements, with an accuracy of 0.9106 ± 0.08, sensitivity of 0.7768 ± 0.17, specificity of 0.9441 ± 0.07, precision of 0.7984 ± 0.22, F-Score of 0.7829 ± 0.18, and AUC of 0.8605 ± 0.11. A comprehensive overview of the performance stands out as the best performing experiment, demonstrating the effectiveness of the DeepVAQ model in accurately classifying VA quality.

**Table 6:** Performance measurements for different experiments

**Experiment** ${\mathbf{e}\mathbf{x}\mathbf{p}}_{({\varvec{i}},{\varvec{j}})}$	**Accuracy**	**Sensitivity**	**Specificity**	**Precision**	**F-Score**	**AUC**	**Loss validation**
${{\text{exp}}}_{(\mathrm{8,3})}$	0.9021 +—0.09	0.7558 +—0.20	0.9388 +—0.07	0.7698 +—0.24	0.7610 +—0.22	0.8473 +—0.13	1.2521 +—1.02
${{\text{exp}}}_{(\mathrm{8,5})}$	0.8915 +—0.11	0.7267 +—0.37	0.9320 +—0.13	0.7391 +—0.28	0.6996 +—0.35	0.8294 +—0.18	1.4008 +—1.90
${{\text{exp}}}_{(\mathrm{8,7})}$	0.8851 +—0.12	0.7111 +—0.36	0.9280 +—0.15	0.7957 +—0.29	0.6939 +—0.33	0.8196 +—0.18	1.4810 +—1.85
${{\text{exp}}}_{(\mathrm{16,3})}$	0.8745 +—0.07	0.6863 +—0.27	0.9215 +—0.03	0.6681 +—0.17	0.6704 +—0.22	0.8039 +—0.14	1.6079 +—1.40
${{\text{exp}}}_{(\mathrm{16,5})}$^a^	0.9106 +—0.08^a^	0.7768 +—0.17^a^	0.9441 +—0.07^a^	0.7984 +—0.22^a^	0.7829 +—0.18^a^	0.8605 +—0.11^a^	1.1441 +—0.86^a^
${{\text{exp}}}_{(\mathrm{16,7})}$	0.9000 +—0.10	0.7512 +—0.25	0.9376 +—0.08	0.7655 +—0.25	0.7490 +—0.25	0.8444 +—0.15	1.2754 +—1.30
${{\text{exp}}}_{(\mathrm{32,3})}$	0.8830 +—0.11	0.7077 +—0.23	0.9269 +—0.07	0.7287 +—0.28	0.7169 +—0.26	0.8173 +—0.16	1.4985 +—1.19
${{\text{exp}}}_{(\mathrm{32,5})}$	0.8915 +—0.11	0.7300 +—0.25	0.9323 +—0.09	0.7582 +—0.29	0.7335 +—0.26	0.8311 +—0.15	1.3840 +—1.30
${{\text{exp}}}_{(\mathrm{32,7})}$	0.9021 +—0.09	0.7560 +—0.19	0.9388 +—0.07	0.7794 +—0.25	0.7635 +—0.21	0.8474 +—0.13	1.2506 +—0.98

^a^ Best classification result

**Fig. 4:**
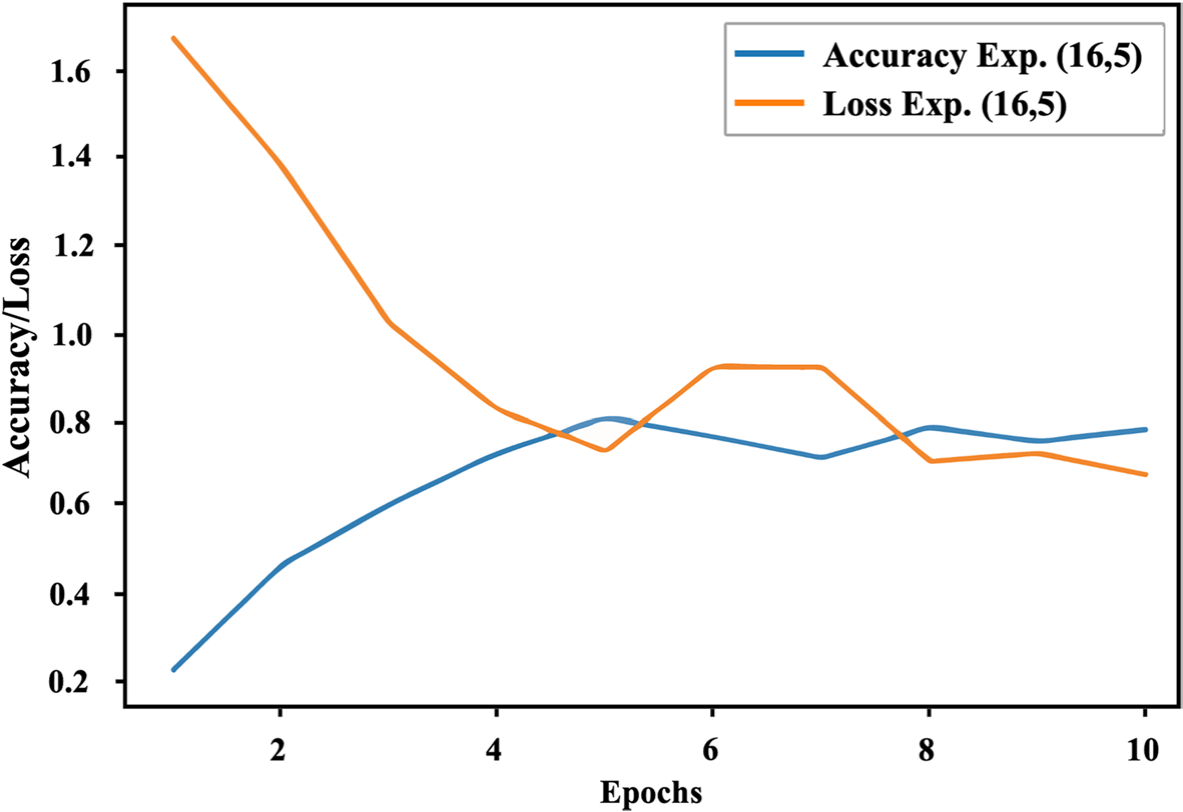
Validation accuracy and loss in ${exp}_{(\mathrm{16,5})}$

Figure 4 illustrates the relationship between the filter sizes and the validation accuracy and loss. It can be observed that as the filter size increases, the validation accuracy improves, reaching a value of 0.8, while the validation loss decreases to 0.7.

The performance of the DeepVAQ model at the configured parameter setting of ${{\text{exp}}}_{(\mathrm{16,5})}$ was evaluated for each VA quality class. The results, summarized in Table 7, demonstrate the accuracy, sensitivity, specificity, precision, and F-Score achieved by the model for each class. For class 1, the model achieved an accuracy of 0.9894, high sensitivity (0.9474) and specificity (1.0000), precise predictions (precision of 1.0000), and a balanced F-Score of 0.9730. Class 4 also had notable results, with an accuracy of 0.9574, sensitivity of 0.8421, specificity of 0.9867, precision of 0.9412, and an F-Score of 0.8889. Additionally, class 5 exhibited exceptional accuracy of 0.9947, sensitivity of 0.973, specificity of 1.0000, precision of 1.0000, and an F-Score of 0.9863. Although classes 2 and 3 had relatively lower accuracies (0.8404 and 0.8245, respectively), the model still demonstrated reasonable performance in differentiating these classes.

**Table 7:** Performance measurements for DeepVAQ model at ${{\text{exp}}}_{(\mathrm{16,5})}$

Approach	Class	Accuracy	Sensitivity	Specificity	Precision	F-Score
**DeepVAQ model**	1	0.9894	0.9474	1.0000	1.0000	0.9730
	2	0.8404	0.5405	0.9139	0.6061	0.5714
	3	0.8245	0.7105	0.8533	0.5510	0.6207
	4	0.9574	0.8421	0.9867	0.9412	0.8889
	5	0.9947	0.973	1.0000	1.0000	0.9863

**Fig. 5:**
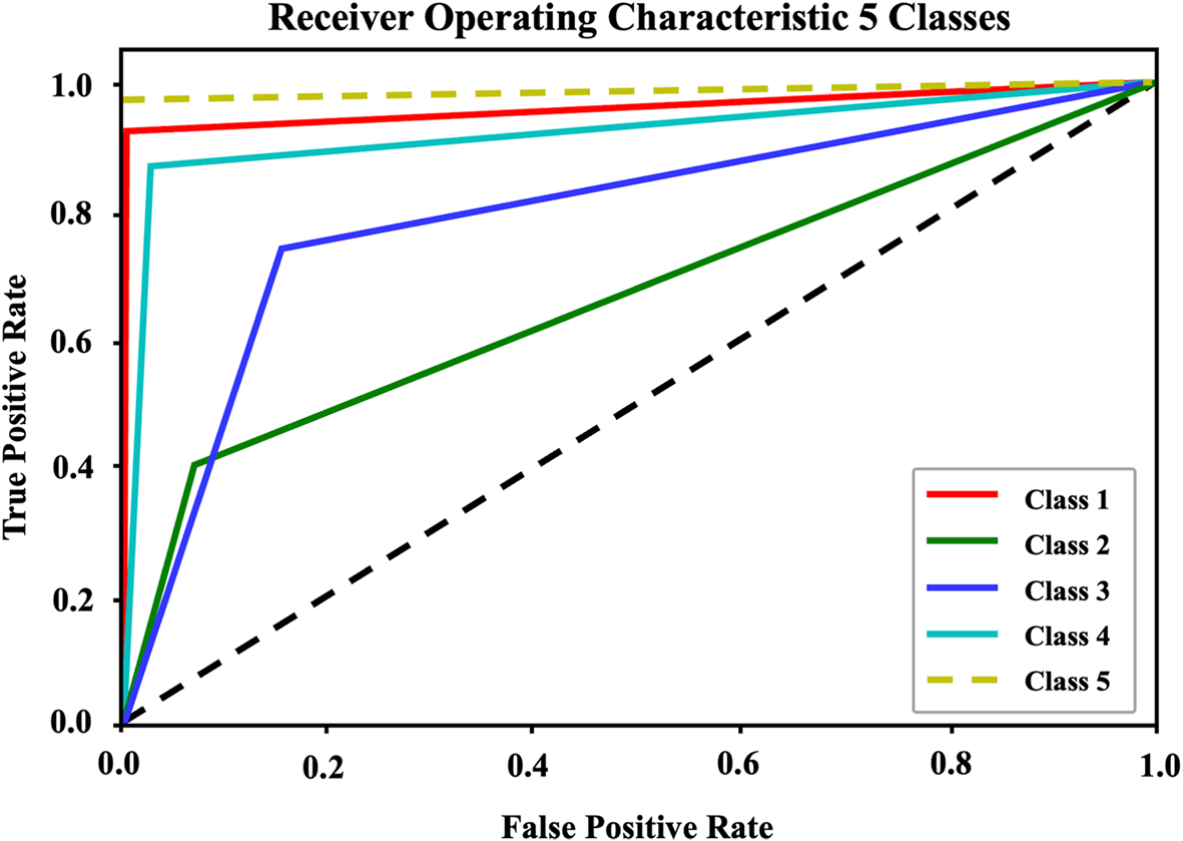
ROC curve for DeepVAQ

To assess the performance of DeepVAQ, we utilized ROC curves and calculated the AUC (Area Under the Curve). Figure 5 illustrates the ROC curve for the DeepVAQ model across multiple classifications. The AUC value provides an indication of the model's predictive capability, with a higher value suggesting better prediction accuracy. In our evaluation, each class achieved a true positive rate exceeding 0.5. The position of the ROC curve closest to the upper-left corner represents a balance between high sensitivity and specificity, indicating an optimal classification performance. This effectively distinguished between different VA quality classes, demonstrating its ability to accurately identify true positives and true negatives.

**Fig. 6:**
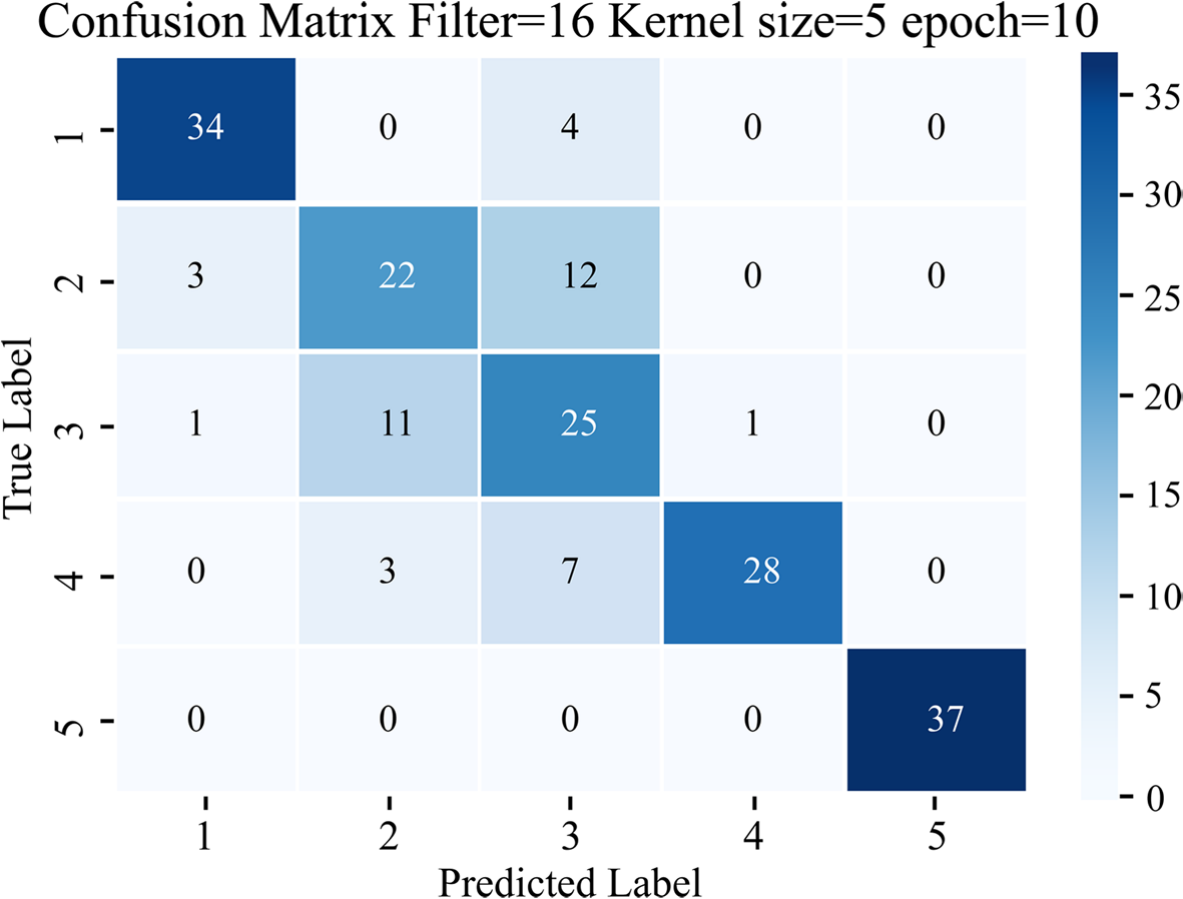
Confusion matrix of DeepVAQ for each class of HD patients

The confusion matrix, displayed in Fig. 6, provides a comprehensive assessment of the proposed DeepVAQ model. It offers valuable insights into the model's performance on the testing data, consisting of 125 records in the test set. The predictions obtained on the test set closely align with the training phase results, highlighting the model's consistency.

Figure 6 illustrates the confusion matrix, where each column represents the assigned VA quality labels, and each row corresponds to the true values. The highlighted colors within the matrix signify correct predictions, demonstrating the model's accuracy in classifying the VA quality classes. Notably, class 1 and class 5 achieved flawless predictions, with all samples correctly identified.

The demonstrates high accuracy in predicting VA quality, particularly in classifying VA quality class 1 as "Poor" and class 5 as "Excellence". Class 1, representing the "Poor" state, indicates a high chance of VA stenosis and thrombosis, making it clinically significant. The model's accuracy in class 5 reflects its ability to identify the highest VA quality. These findings highlight the DeepVAQ model's effectiveness in distinguishing between different levels of VA quality, enabling clinicians to make informed decisions based on the predicted VA quality. The model shows promise in identifying patients at higher risk and assessing overall VA quality.

## Discussion

The empirical evidence presented in this study confirms the DeepVAQ model's exceptional capability in classifying VA quality in HD patients. Our comprehensive comparative analysis, detailed in Table 8, demonstrates DeepVAQ's superior performance metrics against established machine learning models, including Decision Tree [34], Naive Bayes [35], Support Vector Machine (SVM) [36], and k-Nearest Neighbors (kNN) [37]. These findings are not merely academic but carry profound implications for clinical practice. DeepVAQ's high accuracy and precision in non-invasive VA quality monitoring can potentially transform patient management, reducing the reliance on invasive procedures and facilitating proactive healthcare strategies.

**Table 8:** A comparison of prediction results of models

Models	Accuracy	Sensitivity	Specificity	Precision	F-Score
Decision Tree [34]	0.2531	0.1563	0.6550	0.0679	0.3051
Naive Bayes [35]	0.5816	0.3041	0.7159	0.4348	0.2489
k-Nearest-Neighbours (*k*NN) [37]	0.4735	0.2492	0.6912	0.3237	0.1230
DeepVAQ model (A proposed model)	0.9213^a^	0.8431^a^	0.9614^a^	0.8762^a^	0.8364^a^

^a^Best classification result

Acknowledging the limitations of our research, we recognize that the controlled test conditions of our study may not fully encapsulate the complexities of real-world clinical environments. Future studies are required to validate DeepVAQ in diverse clinical settings, ensuring its efficacy across a broader patient population. Additionally, while our model exhibits robustness in classification, the overlap observed between classes 2 and 3 necessitates further refinement of the model's discriminative power. Advanced noise reduction and feature selection techniques, such as Recursive Least Square (RLS) and Least Mean Square Error (LMS), will be pivotal in addressing this challenge.

The potential for AI and machine learning to revolutionize the field of HD patient care is unmistakable. DeepVAQ stands as a testament to this potential, paving the way for integrating such models into routine clinical practice, enhancing patient-centered care, and setting a new benchmark in the management of vascular access.

## Conclusions

The proposed DeepVAQ model demonstrates successful classification of VA quality in HD patients by utilizing multiple PPG sensors. It was developed using a customized hyperparameter setting within the CNN architecture, DeepVAQ achieved superior classification performance when compared to other machine learning models, as evidenced by its highest scores across various performance measurements. This approach offers a non-invasive and cost-effective means of predicting VA quality, which could potentially be accessible to the wider public. The findings highlight the potential of DeepVAQ as a promising tool for early detection of VA quality deterioration in HD patients. By accurately classifying VA quality, this model has the potential to greatly benefit the quality of life for HD patients worldwide. While DeepVAQ performed exceptionally well overall, further enhancements can be explored to improve its ability to differentiate between closely related classes, specifically classes where some overlap was observed.

## Data Availability

The datasets analyzed during the current study is available from the corresponding author on reasonable request.

## Abbreviations

VA: Vascular access
ESKD: End-stage kidney disease
HD: Hemodialysis
VABF-UD: VA blood flow using ultrasound dilution
PPG: Photopletysmography
CNN: Convolutional neural network
DeepVAQ: Deep learning for prediction of vascular access quality in hemodialysis patients
